# Palliative Care for Children in Hospital: Essential Roles

**DOI:** 10.3390/children5020026

**Published:** 2018-02-19

**Authors:** Ross Drake

**Affiliations:** Starship Children’s Health, Park Road, Grafton, Auckland 1023, New Zealand; rossd@adhb.govt.nz

**Keywords:** pediatrics, infant, children, adolescent, hospitals, inpatient, hospital-specific palliative care issues, pediatric palliative care, end-of-life care, life-limiting conditions, chronic disease

## Abstract

Palliative care for children in pediatric hospitals is a vital part of the network of services supporting children with severe illness. This has been recognized, with a trend over the past decade for an increased number of pediatric palliative care (PPC) services established in pediatric hospitals. The inpatient team is in the unique position of influencing the early identification of children and their families, across the age and diagnostic spectrum, which could benefit from palliative care. These services have an opportunity to influence the integration of the palliative approach throughout the hospital, and in so doing, have the capacity to improve many aspects of care, including altering an increasingly futile and burdensome treatment trajectory, and ensuring improved symptom (physical and psychological) management.

## 1. Introduction

Pediatric palliative care (PPC) services have been in development since England led the way with the founding of the first children’s hospice, Helen House, in 1982 and the pioneering hospital-based palliative care team in 1986 at Great Ormond Street Hospital. Internationally, the establishment of PPC services has been variable and largely restricted to countries with a very high human development index. However, the location of these services—whether in the hospital versus the community—has often been imbalanced, with capacity gradually built in the contrasting area over time. For example, the United Kingdom (UK) has had a focus on residential hospice teams, while the United States (USA) has concentrated on hospital-based teams.

The hospital-based PPC service is an important part of the network of care for children with serious illness. The experience of the USA has been detailed in a 2012 survey [1], which indicated a creditable 72% of respondents had a PPC program, with the majority having been launched since 2005. The most common service offered was consultation (88%), with the majority providing this throughout the hospital (87%) and across the age range (>90%). In terms of community capacity, all of the programs reported having developed a relationship with a hospice program, with 80% working with one or more independent hospice organization, and close to 20% working in a hospital that operated its own hospice program.

Regardless of location of care, the philosophy of palliative care remains the same. However, there are some requirements of a hospital-based team over and above that of a community-based service. They hospital service plays an essential role in identifying children and families in need, which includes integrating the palliative approach within the hospital, liaising effectively with community-based services, and providing impeccable assessment and treatment of pain and other problems to the betterment of the mind, body, and spirit of children and their families.

## 2. Identification

Being situated in a children’s hospital, particularly a tertiary facility, means that PPC clinicians are more likely to be exposed to children with rare disorders [2], many of whom have an uncertain or guarded prognosis [3]. This provides an opportunity for the hospital-based service to identify children who could benefit from a palliative approach or palliative care, and relay this to the primary pediatric service engaged in the child’s care. This charges the PPC service to be effective communicators and educators, both formally and informally, to all of the pediatric services, not just the “big three” of oncology, neurology, and cardiology.

The neonatal unit is likely to see the largest number of deaths in a children’s hospital, while the pediatric intensive care unit (PICU) is likely to admit children with a serious illness at their most vulnerable. In a national cohort study [4] of children admitted to a PICU in England over an 11-year period, 57.6% (*n* = 89,127) of admissions and 72.9% (*n* = 4,821) of deaths were of an individual with a potentially life-shortening medical condition. The mortality rate in the PICU for these children was double compared with those who did not have such a diagnosis (5.4% versus 2.7%), and a child with a life-limiting condition was 75% more likely to die in the PICU once all of the factors had been taken into account (OR 1.75; 95% CI 1.64 to 1.87).

*Case 1.* “A” was born by emergency cesarean section at term after placental abruption. She required extensive resuscitation at birth and mechanical ventilation for six days. Magnetic resonance imaging showed severe cerebral edema consistent with severe hypoxic ischemic encephalopathy. She developed seizures, and her electroencephalogram was profoundly abnormal. In discussion with her parents, mechanical ventilation was withdrawn, with the expectation that she would not survive. Surprisingly, she established spontaneous respirations after extubation, and a referral was made to the hospital PPC team. She was seen by the PPC service, and a discussion was held with the family around their goals and expectations of care, and an advance care plan was completed. In line with their wishes, “A” was discharged home once community pediatric support had been arranged. At home, she began feeding orally; she thrived, and medical care was increasingly provided by the developmental pediatric service.

In-depth information on the demographic profile and clinical characteristics of children requiring palliative care has been a relatively recent event. In 2011, a prospective cohort study [5] of 515 children cared for by six hospital-based PPC teams in the United States and Canada was published. The study reported an age range of less than one year (17.3%) to 19 years or older (15.5%), with non-cancer conditions dominating. The single largest group was children with a genetic/congenital condition (40.8%), whereas children with cancer only made up 19.8% of the cohort. The complexity of the care was reflected in 79.6% of children requiring at least one form of long-term medical technology, with 59.6% having a feeding tube of some description (48.5% gastrostomy). The other notable supports were a central venous catheter (22.3%), tracheostomy (10.5%), and 18% having a requirement for assisted respiration.

Similarly, a Malaysian observational analysis by Chong et al. [6] indicated an age spread that was more consistent with general pediatric mortality data, with 44.8% of the 315 children reviewed being one year of age or younger, and 14.6% in the of adolescent age group (13 years to 18 years). The dominant ICD-10 coding identified was similar to the North American study, with 37.1% of children having ‘congenital malformations, deformations and chromosomal abnormalities’, followed by ‘diseases of the nervous system’ (24.1%) and ‘neoplasms’ (19.0%). Again, children with neurological diseases had significantly more physical needs than the other two diagnostic categories (OR = 3.95; 95% CI 1.47 to 10.61, *p* < 0.01).

*Case 2.* “B” was diagnosed at six months of age with a peroxisomal biogenesis disorder, having been hypotonic and troubled by feeding difficulties since birth. She had had a more recent decline in vision and hearing, as well as gross motor and language development. Her liver function tests had become abnormal, with an increased risk of coagulopathy. There was no known treatment for her condition, and while the prognosis was uncertain, it was expected that she would develop progressive white matter degeneration with increasing disability, and ultimately a shortened life expectancy. A referral was made to the hospital PPC service, which transitioned her care to the community, with respite support arranged.

These findings were reflective of a general trend for children admitted to pediatric hospitals having increasingly complex care needs, with attendant high resource use [7,8,9,10]. This data has been further enriched by an elegant, descriptive study [11] of home-based PPC. The most telling finding for these 33 Italian families was the eight hours and 54 minutes per day spent, on average, caring for their child. Feeding was the single most time-consuming activity, at 174 minutes per day, with the additional six hours of care devoted to maintaining an average of five different life-supporting medical appliances. A feeding device was required by 72% of the children, 36% had a tracheostomy, and 55% were on mechanical ventilator support. Care was provided under the duress of tiredness as a result of broken sleep, as caregivers were awake for an average of 67 minutes per night to attend to the child.

## 3. Integration

The diversity of conditions affecting children that could benefit from palliative care and the medically fragile nature of these children requires a hospital-based PPC service to be an integral part of the pediatric hospital. Very little direction exists on how PPC services can achieve such assimilation, although a review on the early integration of palliative care for children with high-risk cancer [12] suggests a model for cancer that could be translated to non-cancer conditions. 

The model addresses three tiers of service delivery within an institution to “function synergistically to maximize early provision”. It proposes a broad institutional palliative approach by supporting primary teams through education and policy initiatives to allow the delivery of core elements of PPC from the time of diagnosis. A middle level identifies specific populations, including children, at the end of life so that they may receive palliative care from the child’s primary pediatric service by way of standardized guidelines or other such management pathways. This leaves specialist palliative care to be available as the third layer to deal with more complex issues, such as difficult symptom management cases or challenging decision-making issues.

The vignette below provides a glimpse into how the early integration of the PPC service and the palliative approach in the tertiary children’s hospital setting can, amongst other things, aid relationship development and parental adjustment, and assist communication and decision-making [13].

*Case 2: Integration.* “B” developed a series of neurological, respiratory, and gastrointestinal complications over the next four years, including recurrent life-threatening respiratory tract infections and compromised liver function. This required frequent hospital attendances, either as an inpatient or for outpatient consultations with her primary pediatric care provided by the pediatric metabolic service. Several other services were also involved including the pediatric neurology, pediatric gastroenterology, and child liaison psychiatry services. In addition, she had a team of allied health practitioners, including a physiotherapist, occupational therapist, and dietician to help support her developmental, nutritional, and equipment needs. These services were guided by hospital policy for a palliative approach, and through an advance care plan, which the family had previously prepared. The plan was reviewed and modified with the family at times of significant changes in the child’s condition. The hospital PPC team maintained contact at outpatient appointments and during admissions, allowing a strong relationship to be established over time, even though the family had moved to another town distant from the pediatric hospital. This allowed the PPC team to address advance care plan reviews, be involved in managing difficult symptoms, and aid communication with the family between services at times of dissonance.

## 4. Symptom Assessment and Management

The reality is that a hospital-based PPC service will be required to deal with the “relief of suffering by means of early identification and impeccable assessment and treatment of pain and other problems” [14] on a relatively frequent basis. At the beginning of the millennium, the sentinel paper by Wolfe et al. exposed the need for a service with a focus on impeccable symptom management in pediatric hospitals [15].

This exploration of parent perception on the end-of-life care of 102 children dying from cancer indicated that 89% of the children were felt to have suffered “a lot” or “a great deal” from at least one symptom in their last month of life, with this most commonly due to pain, fatigue, or dyspnea. The emphasis for the paper, and arguably the most quoted finding, is that treatment was only successful in 27% of children with pain, and 16% of children with dyspnea. However, as important was the finding that suffering from pain was more likely in children where the physician was not actively involved in providing end-of-life care (OR 2.6; 95% CI 1.0 to 6.7).

The success of having such a program based at a children’s hospital was underlined by a prospective study [16] of the symptom burden for 30 children who died as a result of a medical condition in a large, tertiary center. The symptom burden was high, with children having a mean number of symptoms of 11.1 ± 5.6 in the last week of life. Children dying on the ward had significantly more symptoms than those dying in intensive care (14.3 ± 6.1 vs. 9.5 ± 4.7, *p* < 0.02).

Six symptoms, including pain, occurred with a prevalence of 50% or more, and while symptoms were at times distressing, the majority of children were able to be kept “always comfortable” to “usually comfortable” in the last week (64%) and day (76.6%) of life.

*Case 2: Symptom Management.* “B” was readmitted to hospital after further decline in her vision and the development and onset of distressing severe muscle spasms, intractable irritability, gastrointestinal pseudo-obstruction, and gastric bleeding of unknown cause. The PPC service worked in collaboration with the pediatric metabolic, neurology, and gastroenterology services to modify feeding, bowel care, and coagulopathy management. New medications (baclofen, gabapentin, amitriptyline, clobazam) were introduced and titrated to best effect, while non-pharmacological measures (positioning, gastrostomy venting, distraction, and relaxation techniques for parents to use with “B”) were employed to successfully reduce distress, such that her parents were able to contemplate a return home. When she was ready for discharge, several videoconferences were held with the local general pediatric and palliative care teams to ensure a smooth coordinated handover of care. A detailed symptom management plan was drawn up by the palliative care and metabolic teams, and a system for contact was set up so that her parents knew who to call and where to get help.

In 2008, a follow-up study by Wolfe et al. [17] compared the 102 children who died of cancer between 1990–1997 with a follow-up cohort of 119 children who died between 1997–2004 using a similar retrospective parent survey and chart review methodology. The only difference between the two cohorts was the formation of a Pediatric Advanced Care team at the hospital in 1997.

The comparison found no change in the number of children experiencing pain, fatigue, and dyspnea, but reports of “a lot” or “a great deal” of suffering decreased for all of the symptoms in the follow-up group except fatigue. Specifically, parents reporting this level of suffering from pain were down to 47% in the follow-up cohort versus 66% for the baseline cohort (adjusted risk difference 19%, *p* = 0.018), and dyspnea was also lower at 37% versus 58% (adjusted risk difference 21%; *p* = 0.02). Importantly, parents from the follow-up cohort felt “very prepared” for the medical problems experienced by their child at the end of life (56% versus 27%, *p* < 0.001), and for the circumstances at the time of death (49% versus 25%, *p* = 0.002).

The value of a hospital-based PPC service was further reinforced by a retrospective chart review at another major US pediatric hospital, with the analysis of the inpatient deaths of 114 children [18]. This study compared the outcomes of the 25% of children who received a PPC consult with those children who were not seen by the PPC service. The PPC consult group experienced a higher rate of pain assessment during the last 12 to 24 h of admission (adjusted relative risk 1.57; 95% CI 1.16 to 2.10), better documentation around specific actions to manage pain at 12-hourly intervals during the last 72 h of life (adjusted OR 3.14; 95% CI 1.08 to 9.16 to OR 6.51; 95% CI 1.92 to 22.12), and more likely to have a do-not-resuscitate order in place at the time of death (adjusted OR 7.92; 95% CI 2.02 to 31.12).

## 5. Conclusions

Ultimately, these discoveries strongly suggest that the application of the palliative care paradigm and the presence of a pediatric palliative care service in the pediatric hospital is an important enhancement to the palliative care community. Hospital services can aid in the identification of children and their families who could benefit from palliative care, and by way of system integration and the early involvement of a palliative approach, palliative care can help ensure that palliative and dying children are exposed to the minimum amount of suffering by applying, amongst other things, a more aggressive approach to symptom control.
